# Deciphering the role of NETosis-related signatures in the prognosis and immunotherapy of soft-tissue sarcoma using machine learning

**DOI:** 10.3389/fphar.2023.1217488

**Published:** 2023-06-20

**Authors:** Lin Qi, Fangyue Chen, Lu Wang, Zhimin Yang, Wenchao Zhang, Zhihong Li

**Affiliations:** ^1^ Department of Orthopedics, The Second Xiangya Hospital, Central South University, Changsha, China; ^2^ Hunan Key Laboratory of Tumor Models and Individualized Medicine, The Second Xiangya Hospital, Changsha, China; ^3^ Department of General Surgery, Changhai Hospital, Navy Military Medical University, Shanghai, China; ^4^ Department of Microbiology, Immunology, and Molecular Genetics, Long School of Medicine, UT Health Science Center, University of Texas, San Antonio, TX, United States

**Keywords:** soft-tissue sarcoma, NETosis, immune cell infiltration, tumor microenvironment, scoring system

## Abstract

**Background:** Soft-tissue sarcomas (STSs) are a rare type of cancer, accounting for about 1% of all adult cancers. Treatments for STSs can be difficult to implement because of their diverse histological and molecular features, which lead to variations in tumor behavior and response to therapy. Despite the growing importance of NETosis in cancer diagnosis and treatment, researches on its role in STSs remain limited compared to other cancer types.

**Methods:** The study thoroughly investigated NETosis-related genes (NRGs) in STSs using large cohorts from The Cancer Genome Atlas (TCGA) and Gene Expression Omnibus (GEO) databases. The Least Absolute Shrinkage and Selection Operator (LASSO) regression analysis and Support Vector Machine Recursive Feature Elimination (SVM-RFE) were employed for screening NRGs. Utilizing single-cell RNA-seq (scRNA-seq) dataset, we elucidated the expression profiles of NRGs within distinct cellular subpopulations. Several NRGs were validated by quantitative PCR (qPCR) and our proprietary sequencing data. To ascertain the impact of NRGs on the sarcoma phenotype, we conducted a series of *in vitro* experimental investigations. Employing unsupervised consensus clustering analysis, we established the NETosis clusters and respective NETosis subtypes. By analyzing DEGs between NETosis clusters, an NETosis scoring system was developed.

**Results:** By comparing the outcomes obtained from LASSO regression analysis and SVM-RFE, 17 common NRGs were identified. The expression levels of the majority of NRGs exhibited notable dissimilarities between STS and normal tissues. The correlation with immune cell infiltration were demonstrated by the network comprising 17 NRGs. Patients within various NETosis clusters and subtypes exhibited different clinical and biological features. The prognostic and immune cell infiltration predictive capabilities of the scoring system were deemed efficient. Furthermore, the scoring system demonstrated potential for predicting immunotherapy response.

**Conclusion:** The current study presents a systematic analysis of NETosis-related gene patterns in STS. The results of our study highlight the critical role NRGs play in tumor biology and the potential for personalized therapeutic approaches through the application of the NETosis score model in STS patients.

## Introduction

Soft-tissue sarcomas (STSs) are one of the rarest malignancies, making up only about 1% of all cancers in adults (Gamboa et al., 2020). Annually, there are approximately 5 to 6 cases of STSs per 100,000 people (Gage et al., 2019). The STS has a predilection for middle-aged and older adults. Several certain risk factors including gene mutation and exposure to radiation or chemicals have been identified, but the specific etiology of most STS remained unknown (Hoefkens et al., 2016). STSs are extremely heterogeneous that originate from mesenchymal cells, with peripheral extremities as the most prevalent primary sites (von Mehren et al., 2022). It has been a priority to identify new treatments for STS and improve patient outcomes in recent years. As a promising treatment, immunotherapy stimulates the body’s immune system to recognize and attack STS cells (Birdi et al., 2021). However, treatments can be challenging to conduct due to heterogeneous histological and molecular characteristics.

The term NETosis refers to the process about the cell death characterized by neutrophils releasing extracellular structures known as neutrophil extracellular traps (NET), which contain DNA, histones, and antimicrobial proteins that are capable of trapping and killing bacteria and other pathogens (de Bont et al., 2019). The innate immune response of NETosis was initially defined as an anti-infectious effect, but recent studies have suggested that NETosis also contributed to a number of noninfectious pathological conditions, including cancers (Cedervall et al., 2016). Malignancies could induce platelet activation contributing to cancer-related thrombosis, as well as enhanced metastasis through a variety of mechanisms (Gay and Felding-Habermann, 2011). Additionally, it has been reported that neutrophils that form complexes with platelets are associated with organ failure in cancer patients (Cedervall et al., 2015). NETs lead to the poor peripheral perfusion during the cancer, and DNase I could destabilize and remove NETs due to the high extracellular DNA content of NETs. Studies in mouse models have shown that targeting NETs with DNase I reduced tumor growth and improved chemotherapy efficiency (Cools-Lartigue et al., 2013). In addition, the enzyme protein-arginine deiminase 4 (PAD4), mediating the citrullination of histones, has been identified as a key player in the process of NETosis (Garcia-Gerique and Nefedova, 2023). PAD4 could facilitates the NETs releasing by converting arginine residues on histones to citrulline. As a result, PAD4-mediated NETosis were reported to contribute to tumor growth and metastasis, through its modulation of the tumor microenvironment (TME) and promoting angiogenesis (Li et al., 2020). Although the NETosis has emerged as a pivotal factor about the diagnosis and treatment in cancers, researches about STS are still extremely limited compared with other types of cancers.

Herein, our objective was to explore in depth NETosis-related genes (NRGs) within STS by using large cohorts within The Cancer Genome Atlas (TCGA) as well as Gene Expression Omnibus (GEO) databases. Employing a range of machine learning-based algorithms, we systematically screened for NRGs. A comprehensive analysis of genome and transcriptome characteristics of NRGs in STSs was conducted. A number of NETosis-related clusters and subtypes have been identified, and characteristics of TME have been investigated in depth. Furthermore, our study established a NETosis scoring model that could be used to predict the prognosis for STS patients and the response to immunotherapy. Overall, the findings of this study hold the potential to contribute to a better understanding of the biology and treatment for STS.

## Methods

### Data sources and processing

The TCGA and GEO datasets were utilized to gather gene expression profiles and clinical information of STS. Using the Genotype-Tissue Expression (GTEx) database, we extracted gene expression matrix for normal adipose and muscle tissues. For the comparison of the gene expression profiles of TCGA and GTEx datasets, UCSC Xena has utilized rigorous analyze pipeline (TOIL RNA-seq). This algorithm could enable the direct comparison between tumor and normal tissues at the gene expressing level (Wang et al., 2018). We obtained all data from the TCGA using UCSC Xena browser, including mutations frequency, variability in somatic copy number (SCNV) and RNA-seq data. A comprehensive pan-cancer investigation was conducted using data from the TARGET Pan-Cancer (PANCAN) cohort. We also screened out two cohorts (GSE17118 and GSE30929) that included prognosis data, as well as a single-cell RNA-seq (scRNA-seq) dataset for STS (GSE131309), by using the GEO database. In addition, our study included the gene matrix and clinical information of patients receiving a combination therapy of PD-1 and CTLA-4 blockade (Gide et al., 2019).

### Unsupervised clustering of NRGs

Based on research of NETosis in different diseases (Şenbabaoğlu et al., 2016; Papayannopoulos, 2018; Zhang et al., 2022), gene sets of NETosis have been summarized (Supplementary Table S1). By utilizing the “glmnet” package, we performed Least Absolute Shrinkage and Selection Operator (LASSO) regression analysis and tenfold cross-validation to determine the penalty regularization parameter *λ*. As another machine learning method, support vector machine recursive feature elimination (SVM-RFE) adopts the structural risk minimization principle and aims to optimize the learning performance by minimizing the empirical error. To visualize genomic location of NRGs, we used the “Rcircos” package (version 1.2.1) to plot the chromosomes in a circular pattern. The STS patients were grouped into specific clusters and subtypes based on unsupervised clustering of NRGs. For enhancing the stability of clustering, we employed the R package “ConsensusClusterPlus” with optimal parameters set as maxK of 9 and repetitions of 1,000 (Wilkerson and Hayes, 2010).

### Gene set variation analysis (GSVA)

For the purpose of investigating the biological characteristics of different clusters, subtypes and low and high NETosis scoring groups, we conducted following GSVA, by utilizing the R package “GSVA” (Hänzelmann et al., 2013). The Molecular Signatures Database (MSigDB) provided predefined gene datasets (h.all.v7.5.1). In order to analyze the enrichment scores, we utilized the outputs of GSVA through R package “limma”. The modified t-statistics were then used to analyze the data. By utilizing the R package “ggplot2″, the outcomes were further visualized. The “clusterProfiler” R package was also utilized for detecting significant enrichments, based on GO annotations with the threshold of false discovery rate (FDR) < 0.05 (Yu et al., 2012). We visualized the correlation between NRGs in STS with the Spearman correlation test, by using the R package “corrplot”. The interactive network illustrating the relationship between NRGs and prognostic data was created by utilizing the R package “igraph".

### Detection of differentially expressed genes (DEGs) between clusters

After identifying the NETosis-related clusters through unsupervised clustering, we conducted the analysis of DEGs using the “limma” R package. The functions of “lmFit” and “eBayes” were employed to guarantee precision. For addressing the issue of multiple comparisons, we applied the Benjamini–Hochberg method to adjust the *p*-values. We then filtered DEGs with an adjusted *p*-value <0.05.

### Quantification of immune infiltration in TME

The infiltration of specific groups of immune cell groups was assessed using single-sample gene set enrichment analysis (ssGSEA). The previously published research provided gene signatures for specific clusters of immune cells used in ssGSEA (Bindea et al., 2013). A scale ranging from 0 to 1 was utilizing for normalizing immune cell infiltrations. We also utilized previously established signatures of tumor mutation burden (TMB), to explore the relationship between TME and potential biological processes (Mariathasan et al., 2018). The ESTIMATE scores were calculated using the R package “ESTIMATE” to estimate stromal and immune infiltration in the samples based on gene signatures (Yoshihara et al., 2013). From previously published studies, we also retrieved signatures associated with pathways predicted to be involved in immunotherapy response and cancer-immunity cycles (Chen and Mellman, 2013; Qi et al., 2023). The framework of cancer-immunity cycles guides cancer immunotherapy (Chen and Mellman, 2013). The approaches for computing the activity of these steps were described in a previous publication (Xu et al., 2018). Using the R package “ggcor”, we further evaluated and compared the association between the NETosis scores and the GSVA scores of the signatures mentioned above.

### Development of NETosis score

In order to score activity of NETosis, the following system was developed. Initially, unsupervised clustering has been used for identifying unique NETosis-related clusters. Following this, we screened and selected DEGs overlapped between clusters. Next, we performed a principal component analysis (PCA) using these DEGs. In order to calculate NETosis scores, we selected both PC1 and PC2 from the PCA of above DEGs. As a result of this method of scoring, the dataset for most well-correlated (or anticorrelated) genes will be given a higher score, while genes that are not related to most set factors will be given a lower weight, as they have been implemented in previous investigations (Zhang et al., 2020; Chong et al., 2021). The scoring system to evaluate NETosis score was computed using the subsequent equation: NETosis score = Σ (PC1_*i*_ + PC2_*i*_), where *i* indicates the expression of the selected DEGs based on the PCA. An algorithm-derived optimal cut-off value was further introduced to classify patients with STS into high and low NETosis groups.

### Transcriptome analysis at single-cell level

Data from a previously published scRNA-seq research (GSE131309) were used for this study (Jerby-Arnon et al., 2021). According to the standard pipelines, we used the Seurat package to analyze the scRNA-seq data. The quality control (QC) metrics as well as other parameters used, were in accordance with those in the publications (Jerby-Arnon et al., 2021; Qi et al., 2022). Furthermore, we annotated specific cell clusters using the same labeling system as the original study, and the corresponding annotation methods were described in detail in that study (Jerby-Arnon et al., 2021). Visualization of expression patterns of NRGs were also conducted at the single-cell resolution.

### Predicting chemotherapy sensitivity

The Genomics of Drug Sensitivity in Cancer database (GDSC), was used to collect information on drug response. (Yang et al., 2013). There are over 1,000 human cancer cell lines in the GDSC database along with 518 compounds that target 24 pathways. We utilized the R packages “pRRophetic” and “oncoPredict” to compute the IC50 and drug sensitivity score (Iorio et al., 2016; Maeser et al., 2021).

### Long-read RNA sequencing

The expression levels of NRGs were verified using our own sequencing data, which consisted of four pairs of STS and paired normal tissues (GSE198568). The long-read RNA sequencing was conducted by Biomarker Technologies (Beijing, China), in compliance with the standards by Oxford Nanopore Technologies.

### Cell lines and quantitative PCR (qPCR)

Information about the sources for the cell lines of sarcoma including SW-982, SW-872, hSS-005R and HSF used in this study was provided in previous publications (Qi et al., 2022; Qi et al., 2023). In order to cultivate the cell lines of STS, Dulbecco’s modified Eagle medium (DMEM) supplemented with 10% fetal bovine serum (FBS) were used at the temperature of 37°C with 5% CO_2_ atmosphere.

For qPCR, we used the RNA Express Total RNA Kit (M050, NCM Biotech, China) for extracting total RNA from cell lines. The RevertAid First Strand cDNA Synthesis kit (K1622, Thermo Fisher Scientific, United States) was used to synthesize cDNA. The qPCR refers to the previous studies (Qi et al., 2022; Qi et al., 2023). The primer sequences used for qPCR were summarized in Supplementary Table S2.

### Cell transfection

We obtained siRNAs targeting negative control (NC) and HMGB1 via Hanbio (Shanghai, China). Upon reaching 50% confluency in the 6-well plate, hSS-005R were transfected with 50 nmol of NC and HMGB1 siRNAs with 5 μL Lipofectamine 2000 reagent for 12 h. Supplementary Table S1 illustrated the sequence of siRNAs used in this research.

### Cell proliferation assay

The viability evaluation of the hSS-005R was conducted using the cell counting kit-8 (CCK-8). We seeded the hSS-005R cells in 96-well plates with 2000 cells per well and then incubated the plates overnight. Following transfection, hSS-005R cells were cultured for the indicated durations. Each well received 10 μL of CCK-8 solution followed by 90 μL of DMEM supplemented with 10% FBS at every time point. After 1.5 h of incubation, the optical absorbance at 450 nm was recorded using a microplate reader.

### Clone formation assay

Following transfection, we seeded hSS-005R cells in 6-well plates with 1,000 cells per well and cultured them for 2 weeks, to conduct clone formation assay for assessing cell proliferation. We fixed the cells in 4% paraformaldehyde (PFA) for 15 min and stained them with 0.2% crystal violet for anothewr 15 min.

### Wound healing assay

In order to assess migration capacity, the wound healing assay was conducted. Upon reaching 70% confluence, hSS-005R cells were transfected into various 6-well plates. After reaching 100% confluence, hSS-005R cells were subjected to wound healing assays by creating a scratch using a 100 μL pipette tip. The DMEM with 2% FBS was then used to culture the wounded cells following washing with PBS. We further measured the area covered by the migrated cells after 0 and 48 h, by using the light microscope.

### Statistical analysis

We conducted data analysis using the R software (version 4.1.0). In order to determine whether NRGs are correlated, the Spearman correlation test was performed. Parametric and nonparametric comparisons were conducted for pairwise comparisons using Student’s *t*-test or Wilcoxon signed-rank test, respectively. Likewise, One-way ANOVA and Kruskal–Wallis tests were introduced for analyzing more than two groups. The survival curves were compared using the log-rank test. For the purpose of identifying significant prognostic factors, univariate and multivariate Cox regression analyses were performed. To determine the most appropriate cutoff values for NETosis scores across datasets, the “surv_cutpoint” function from the “survminer” package was repeatedly applied. The NETosis scores of the patients in the datasets were then used to divide them into subgroups of low and high scores. Also, comparing clinical characteristics between two groups was performed using the Chi-square test or Fisher’s exact test. Statistical significance was defined as a *p*-value less than 0.05 with two tailed tests.

## Results

### Selection for candidate NRGs

**FIGURE 1:**
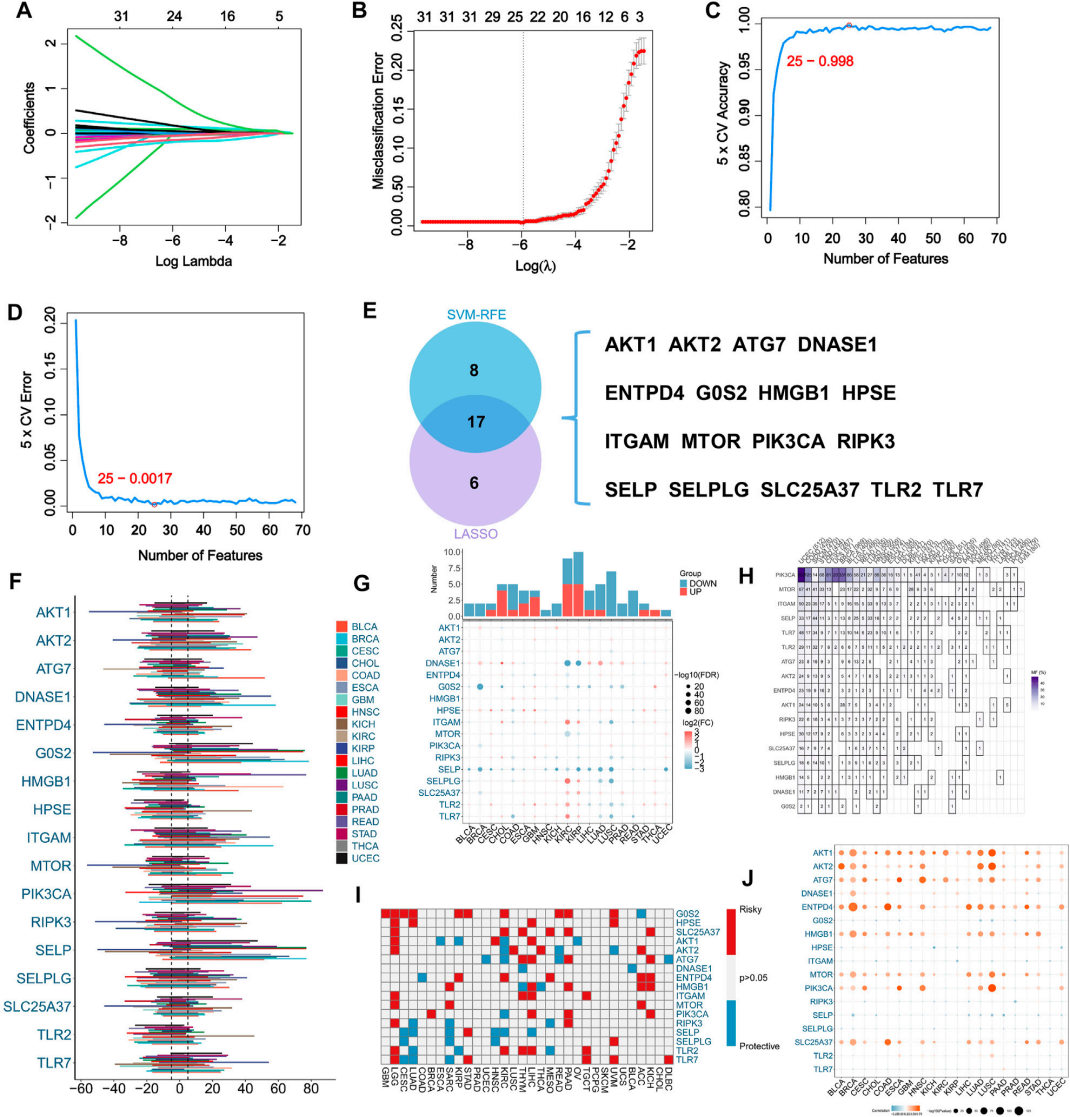
Machine learning assisted NETosis-related genes (NRGs) identification and pan-cancer analysis. **(A)** LASSO coefficient profiles of the 69 NRGs. **(B)** LASSO cross‐validation to select optimal tuning parameter (λ) from the 69 NRGs. **(C)** The accuracy and error **(D)** rate of estimate generation in SVM‐RFE algorithm for selecting NRGs. **(E)** The overlapping of identified NRGs through two machine learning algorithms. **(F)** Variability in somatic copy number (SCNV) of NRGs across different cancer types in TCGA pan-caner cohort. **(G)** Comparison of expression levels of NRGs between tumors and corresponding normal tissues in TCGA pan-caner cohort. **(H)** Variations in NRG mutation frequency among cancer types in TCGA pan-caner cohort. **(I)** Prognostic roles of NRGs in different types of cancer in TCGA pan-caner cohort. Factors with poor prognoses are represented by red, and factors with favorable prognoses are represented by blue. **(J)** Correlation analysis between NRG expression and SCNV in various cancer types.

A total of 69 NRGs have been identified based on comprehensive analysis of the existing literatures on the disease. For the purpose of identifying potential NRGs, we used two different machine learning-based algorithms. The LASSO regression analysis identified 23 potential NRGs from 69 genes with optimal performance (Figures 1A, B). When the number of features was reduced to 25, SVM-RFE achieved minimum error (Figures 1C, D). Comparing the results from the two algorithms above, Venn diagrams were used to identify 17 intersecting NRGs (Figure 1E).

### Pan-cancer analysis of NRGs

Our initial investigation focused on the pan-cancer profile of NRGs. As a result of SCNV of NRGs in pan-cancer, we found SCNV gain in DNASE1, HMGB1 and PIK3CA (Figure 1F). Moreover, comparing normal samples with cancer samples, SELP was downregulated in most types of cancer (Figure 1G). Among the 17 analyzed NRGs in various types of cancer, mutations of PIK3CA were prevalent in a variety of cancer types (Figure 1H). Additionally, G0S2 and SLC25A37 were identified as risk factors for multiple types of cancer (Figure 1I). Besides, it has been found that SCNV and expression of AKT1, AKT2, ATG7, ENTPD4, HMGB1, MTOR, PIK3CA and SLC25A37 were significantly correlated (Figure 1J).

### Genome and transcriptome characteristics of NRGs

**FIGURE 2:**
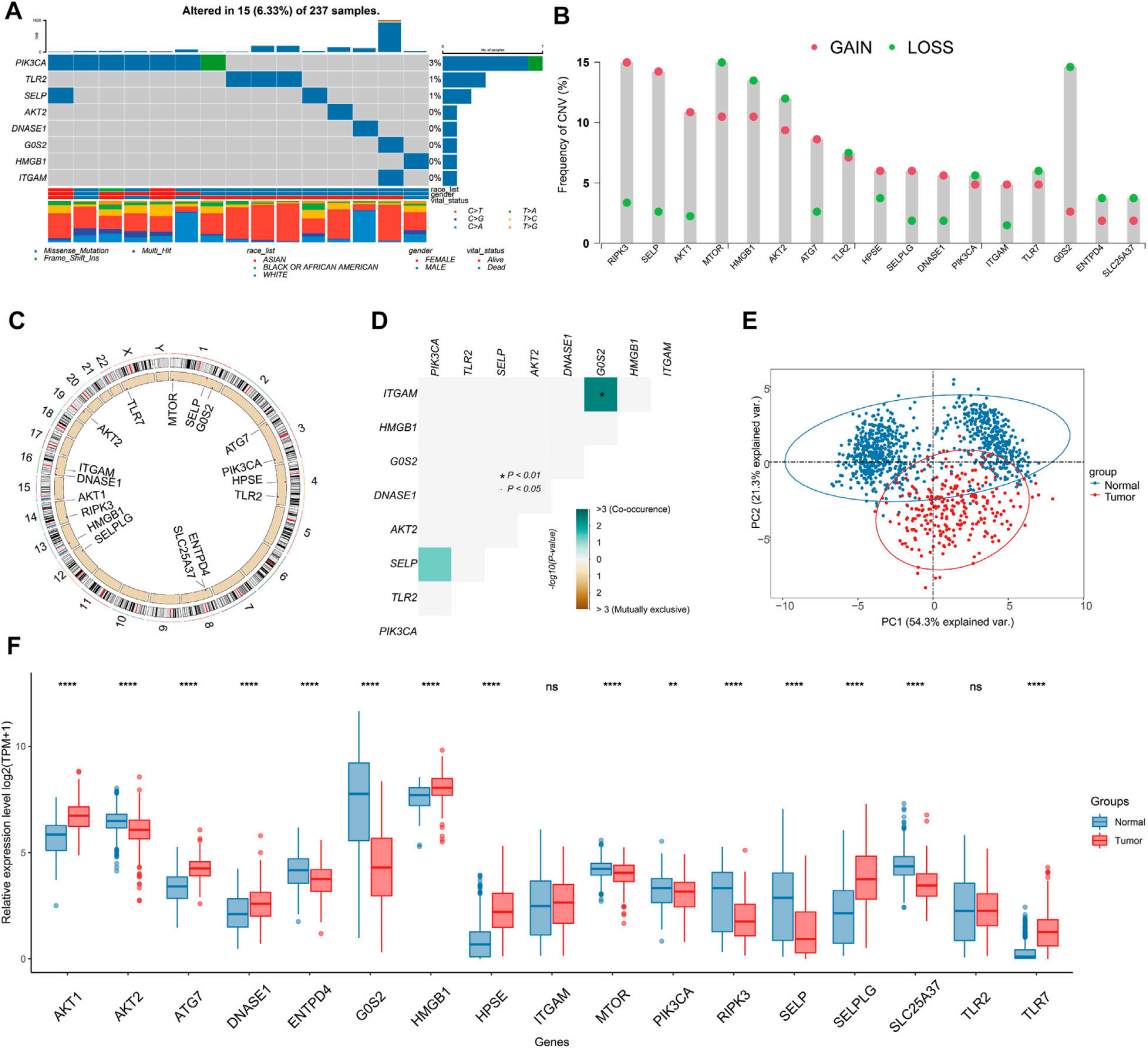
Genome and transcriptome characteristics of NRGs. **(A)** Mutation frequencies of NRGs in TCGA-SARC cohort (Top 8). **(B)** Gain and loss in CNVs of NRGs in the TCGA-SARC cohort. **(C)** Overview of the location of NRGs on human chromosomes. **(D)** Analysis of the mutation co-occurrences and exclusions among mutated NRGs. **(E)** Principal component analysis (PCA) of NRG expression to differentiate soft-tissue sarcoma (STS) from normal tissues. **(F)** Comparison of expression levels of selected NRGs between STS and normal tissues using the TCGA-GTEx database.

Within the TCGA-SARC cohort, we found that merely 6.33% (15 out of 237) of samples present mutations linked to NRGs, and that these mutations were concentrated within 8 specific NRGs (Figure 2A). The frequency of SCNV in NRGs is illustrated in Figure 2B, with RIPK3 showing the highest gain. The majority of NRGs were found to be located on chromosomes 1, 4, 8, and 16 (Figure 2C). By using the somatic interaction function, we also investigated the interactions among somatic mutations of NRGs. Our findings suggested that ITGAM exhibited co-occurrence with G0S2 (*p* < 0.01) (Figure 2D). It is noteworthy that 17 NRGs were able to differentiate between tumors and non-tumor tissues through expression profiling (Figure 2E), as a significant differential expression was observed in the majority of NRGs (Figure 2F).

**FIGURE 3:**
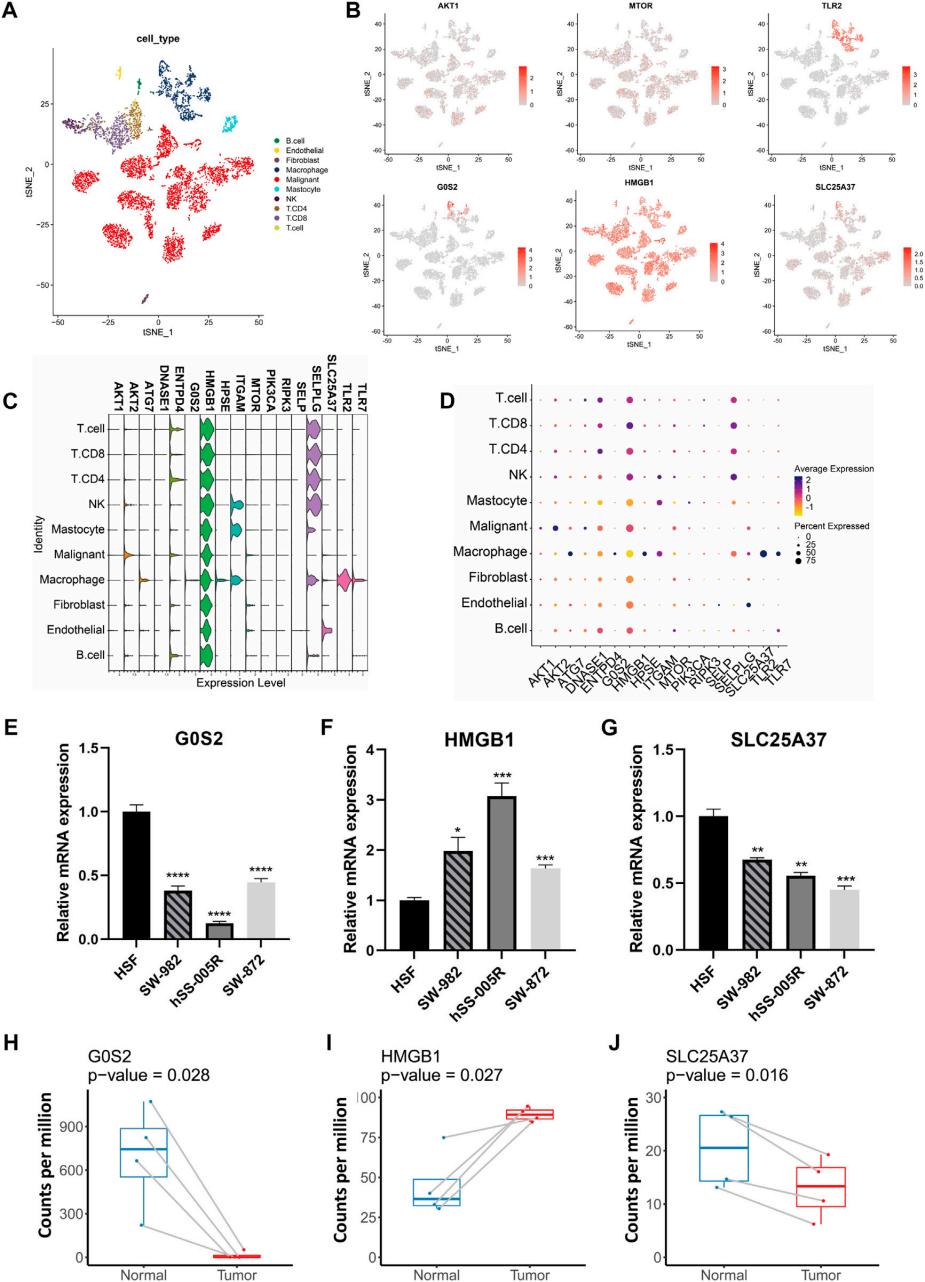
The expression patterns of NRGs illustrated by scRNA-seq data and validation in cell lines. **(A)** The t-distributed stochastic neighbor embedding (t-SNE) of annotated cell types in GSE131309. **(B)** Differences in the expression patterns of specific NRGs between cell types. **(C)** Stacked violin plots illustrating the levels of expression of specific NRGs in different cell types. **(D)** Percent of cells expressing specific NRGs and corresponding expression level. **(E–G)** Quantitative PCR validation of NRG expression in STS cell lines. **(H–J)** Comparison of NRG expression levels between STS and adjacent normal tissues using own sequencing data.

Our analysis of scRNA-seq data from GSE131309 allowed us to further investigate the expression patterns of NRGs (Figures 3A, B). Interestingly, HMGB1, AKT1 and MTOR were found to be expressed across all types of cells, whereas TLR2 and G0S2 were predominantly found in specific cell clusters (Figures 3B–D; Supplementary Figure S1). Validation of qPCR showed that the HMGB1 was significantly higher within sarcoma cell lines, such as SW-982, hss-005R, and SW-872, when comparing with the HSF cell line. By contrast, STS cell lines expressed lower levels of G0S2 (Figures 3E–G). Similarly, A similar pattern of consistency was found within our own sequencing dataset of four pairs of STS with matched normal tissue samples (Figures 3H–J).

### NETosis-related clusters and interactions

**FIGURE 4:**
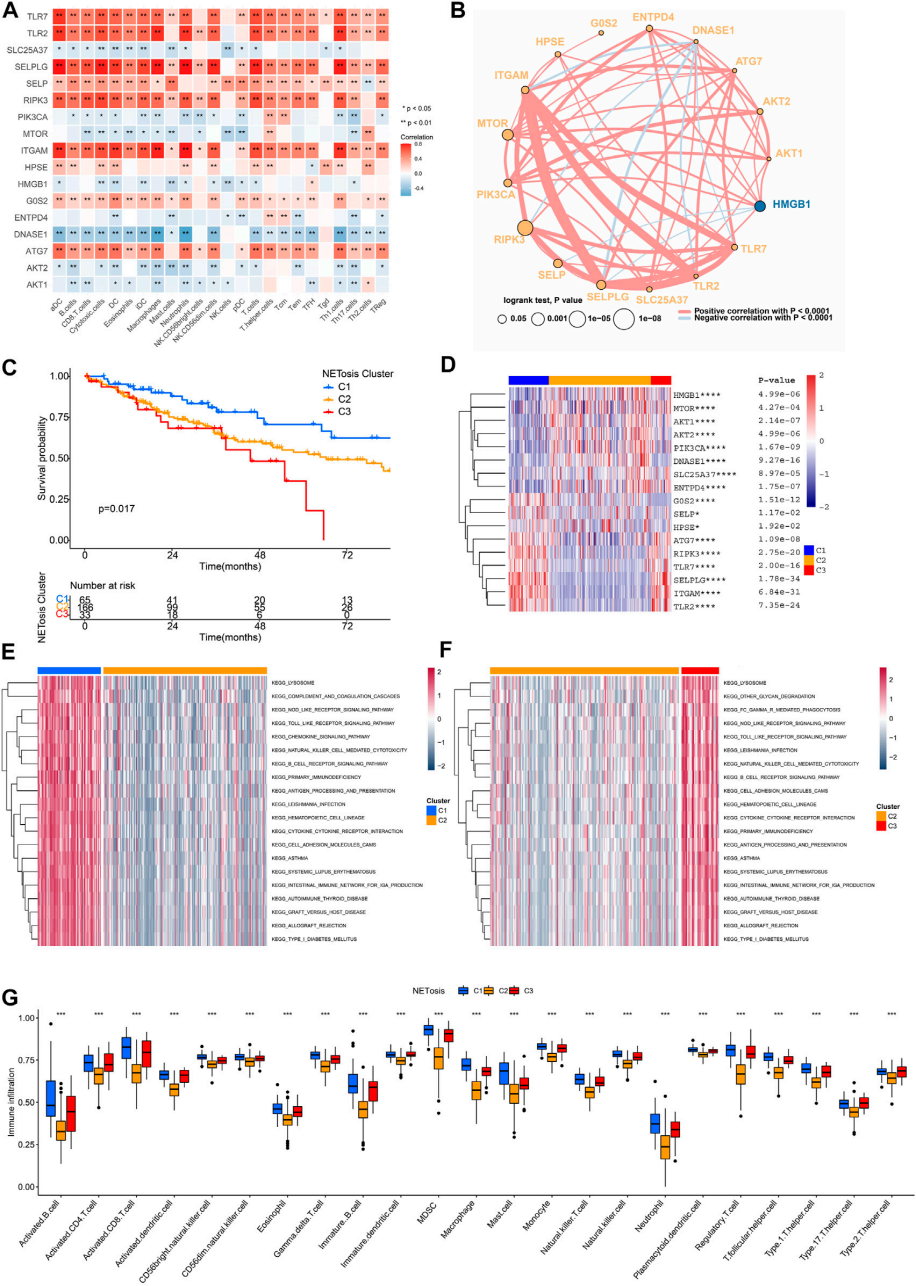
NETosis-related clusters and interactions between NRGs. **(A)** Correlation analysis of NRG expression and immune cells signatures in TCGA-SARC cohort. **(B)** Cross-talk of NRG network TCGA-SARC cohort. **(C)** Kaplan-Meier survival curve of various NETosis-related clusters. **(D–F)** The gene set variation analysis (GSVA) revealing distinct enriched pathways in specific NETosis-related clusters. **(G)** Comparison of immune cell infiltration between different NETosis-related clusters.

As immune cells could interact with other cell types and influence their fate, the TME is essential to the regulation of tumor progression. Upon analyzing relationships among expression levels of NRGs as well as immunocyte signatures, we discovered that TLR7, TLR2, SELPLG, SELP, RIPK3, ITGAM and ATG7 were positively correlated (Figure 4A). A comprehensive overview of the interactions was also constructed by the network of 17 NRGs (Figure 4B). Most NRGs were positively correlated. It is noteworthy that DNASE1 exhibited a negative correlation with a significant number of NRGs.

Using unsupervised consensus clustering, TCGA-SARC cohorts were grouped based on the expression of 17 NRGs (Supplementary Figures S2A–F). As a result, the optimal clustering number was found to be *K* = 3, including 65 cases in C1, 166 cases in C2 and 33 cases in C3, accordingly. The survival rates among the three distinct clusters were significantly different, with C1 showing a noticeable advantage in survival (Figure 4C). There expression patterns of 17 NRGs were illustrated in Figure 4D. As a result of GSVA, we were able to compare the pathway enrichment among clusters (Figures 4E, F). In the C1 cluster, there was a significant enhancement observed in the lysosome, chemokine signaling pathway, and B cell receptor signaling pathway. In addition, the ssGSEA analysis demonstrated that C1 was characterized by the infiltration of innate and adaptive immunocytes (Figure 4G; Supplementary Figure S2G).

### Identification of NETosis-related subtypes

**FIGURE 5:**
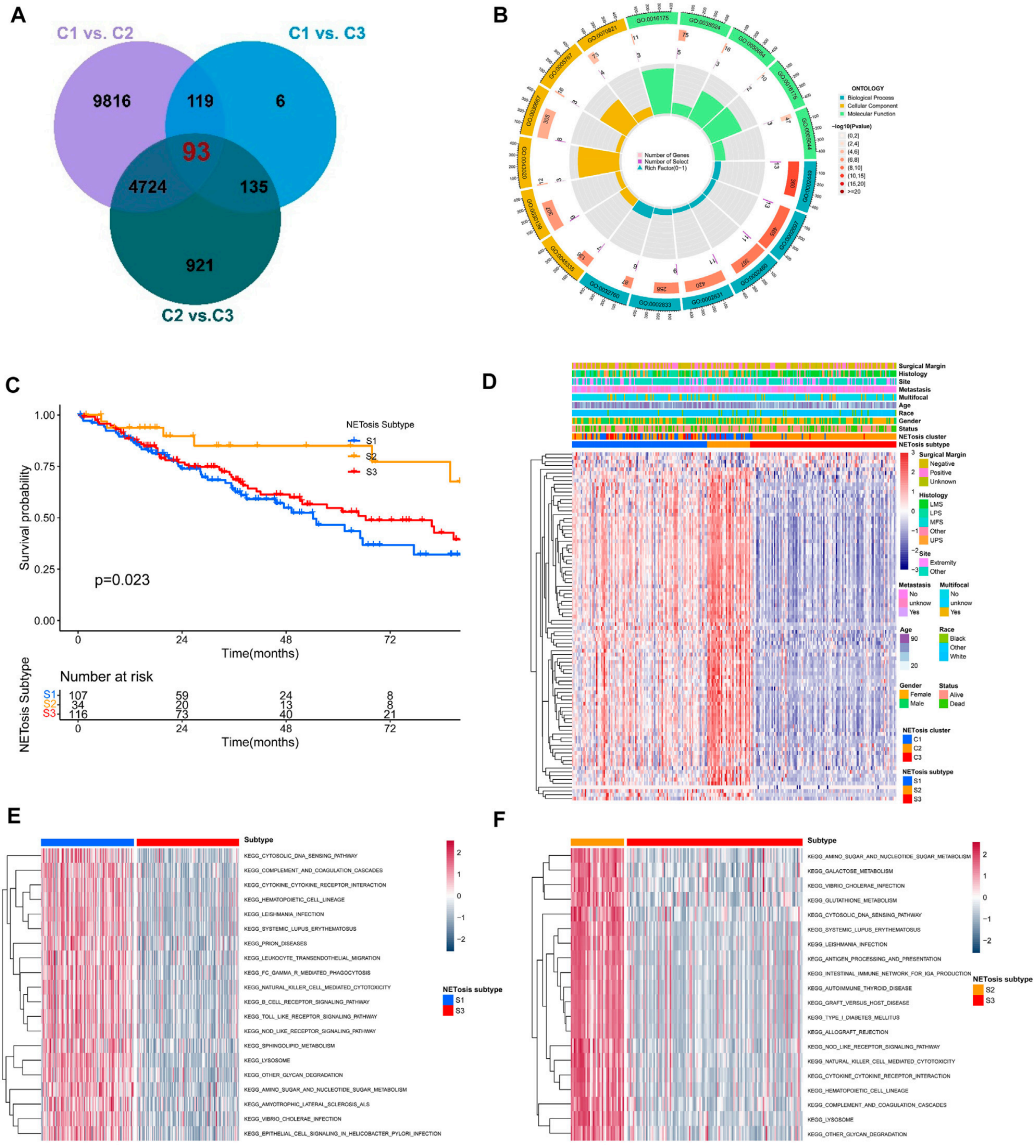
Identification of NETosis-related subtypes and corresponding biological role. **(A)** Venn diagrams showing the overlaps of differentially expressed genes (DEGs) between NETosis-related clusters. **(B)** The Gene Ontology (GO) of overlapped DEGs. **(C)** Kaplan-Meier survival curve of different NETosis-related subtypes. **(D)** Heatmap showing the unsupervised clustering of NETosis-related subtypes in relation to overlapped DEGs. **(E, F)** Comparison of the GSVA illustraring different enriched pathways for NETosis-related subtypes.

We further identified totally 93 DEGs associated with NETosis-related clusters, which were used to investigate the clinical and biological features of clusters (Figure 5A). Furthermore, DEGs were predominantly enriched within GO terms, including lymphocyte mediated immunity, immune effector regulation, and biotic response regulation (Figure 5B). For identifying unique STS cohorts by features of NETosis-related clusters, the unsupervised consensus clustering was conducted by utilizing the above mentioned DEGs (Supplementary Figure S3A–F). Consequently, we identified three distinct subtypes (S1, S2, S3) containing respective patient counts of 107, 34, and 116. The survival outcomes for patients within these subtypes exhibited significant differences (Figure 5C). Despite their distinct gene expression profiles, the clinical features of the three NETosis-related subtypes were more heterogeneous (Figure 5D). GSVA indicated the significant enrichment of cytosolic DNA sensing pathway, cytokine receptor interaction and amino sugar and nucleotide sugar metabolism in S1 subtype (Figure 5E). Intriguingly, in contrast to subtypes S1 and S3, subtype S2 exhibited a greater enrichment within antigen processing and presentation (Figure 5F; Supplementary Figure S3E).

### Development and verification of NETosis score

**FIGURE 6:**
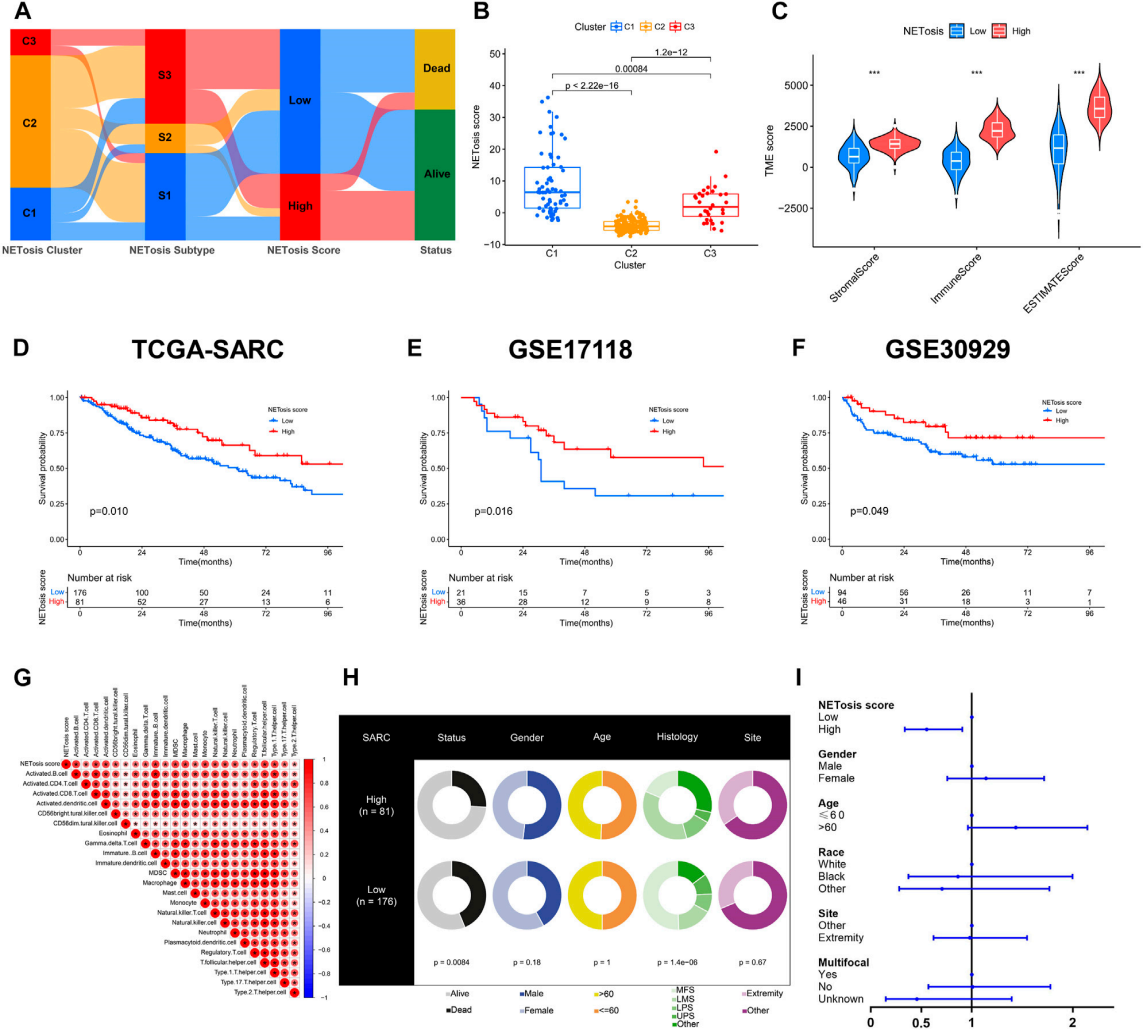
Developing and validating the NETosis score. **(A)** The alluvial diagram depicting the correlation between NETosis-related clusters, subtypes, scores, and survival rates. **(B)** Overview of the NETosis scores of different NETosis-related clusters shown by box plots. **(C)** Tumor microenvironment (TME) scores based on ESTIMATE algorithm between low and high NETosis score groups. **(D–F)** Kaplan-Meier survival curve to validate the prognostic value of NETosis scores in TCGA-SARC, GSE17118 and GSE30929 cohorts. **(G)** Correlation analysis of immune cell signatures and NETosis scores. **(H)** Pie plots showing differences in clinical characteristics between groups with low and high NETosis scores. **(I)** Forest plot illustrating the multivariate Cox regression analysis of NETosis scores and clinical characteristics.

In spite of identification of specific clusters and subtypes associated with NETosis, these works were limited to the TCGA-SARC cohort. Utilizing NETosis-related DEGs, we subsequently constructed a model for NETosis score calculation in STS patients that could be tailored to their individual needs. This illustration presented the NETosis score developmental alluvial diagram and the relations between NETosis-related clusters, subtypes and scores (Figure 6A). Notably, NETosis-related clusters indicated significant differences in NETosis scores (Figure 6B). Afterwards, an optimal cut-off value derived by an algorithm was introduced to classify patients with STS into high and low NETosis groups. Further, we observed significant differences between groups with low and high NETosis scores in TME scores, which comprise stromal scores, immune scores, and ESTIMATE scores (Figure 6C). The TCGA-SARC cohorts suggested an unfavorable prognosis for patients with low NETosis scores (*p* = 0.010) (Figure 6D). Validation with external data from GSE17118 (*p* = 0.016) and GSE30929 (*p* = 0.049) further corroborated this finding (Figures 6E, F). Remarkably, several types of innate and adaptive immune cells were positively correlated with NETosis scores, including macrophage, monocyte, B cell, and different T cell subtypes (Figure 6G). There were distinct clinical characteristics among STS patients classified as high and low NETosis scores, including prognosis (*p* < 0.001) and histology (*p* < 0.001) (Figure 6H). The analysis of the multivariate Cox regression model revealed that high NETosis scores were significant prognostic risk factors for STS (Figure 6I).

### Biological features associated with NETosis scores

**FIGURE 7:**
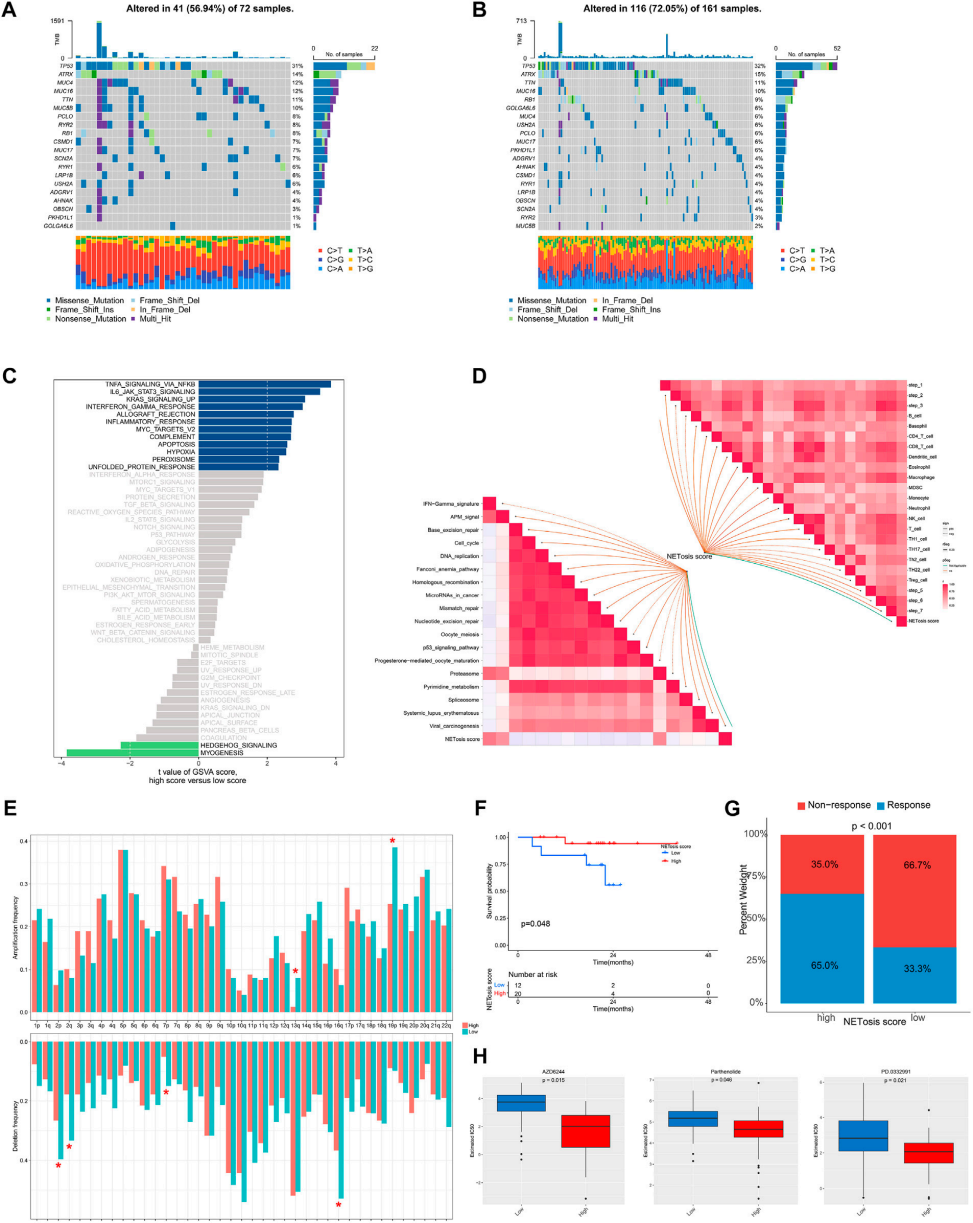
Genomic and transcriptional features associated with NETosis scores in the TCGA-SARC cohort. **(A, B)** Frequently top mutated genes between groups with low and high NETosis scores. **(C)** Bar plot illustrating the differences in enriched pathways by GSVA between groups with low and high NETosis scores. **(D)** Analysis of the correlation of NETosis scores with immunotherapy-predicted pathways and cancer immunity cycles. **(E)** Differences in arm-level amplifications and deletions between low and high NETosis scores. **(F)** Kaplan-Meier survival curve showing the difference between low and high NETosis scores within the cohort treated with immunotherapy. **(G)** The difference in percentage of clinical response of the immunotherapy cohort with low and high NETosis scores. **(H)** Drugs with significantly different estimated IC50 in the TCGA-SARC cohort by NETosis score.

Subsequently, we examined the distinctions in genomic and transcriptomic profiles between high and low NETosis score categories. An increased mutation frequencies was noted in the low NETosis score group, with changes in 116 (72.05%) of the 161 patients (Figure 7A), as opposed to the high NETosis score group, where mutations were observed in 41 (56.94%) of the 72 patients (Figure 7B). It is noteworthy that the prevalence of arm-level amplifications as well as deletions appears more pronounced within the group with high NETosis score relative to the low-score group (Figure 7E). Upon evaluating the enriched pathways in distinct NETosis score groups, we discovered positive enrichment of pathways such as TNF signaling via NF-kB, IL-6/JAK/STAT3 signaling and KRAS signaling in the high NETosis scoring group. Conversely, terms including myogenesis and hedgehog signaling pathways, were observed to be negatively enriched (Figure 7C). Further, we examined the relationship between NETosis scores and immunotherapy-predicted pathways as well as cancer immunity cycles. The NETosis score exhibited a considerable positive correlation with a range of innate and adaptive immune cells, including B cell, different T cell subtypes, dendritic cell, macrophage and others (Figure 7D). Besides, the NETosis scores also demonstrated a positive correlation with several immunotherapy-predicted pathways, including IFN-γ signature and proteasome.

A further analysis of the NETosis score within a cohort receiving immunotherapy was conducted for exploring the correlation among NETosis score and immune status. It is noteworthy that patients displaying low NETosis scores exhibited unfavorable survival outcomes (*p* = 0.048) (Figure 7F), as well as poor response to immunotherapy (*p* < 0.001) (Figure 7G). By screening the GDSC database, we identified drugs that exhibit various response patterns between groups with distinct NETosis scores. A significant difference was noted in IC50 values between the high NETosis-score group and the low NETosis-score group with regard to AZD6244 (*p* = 0.015), Parthenolide (*p* = 0.046), and PD.0332991 (*p* = 0.021).

### Effects of NRGs on STS cell line

**FIGURE 8:**
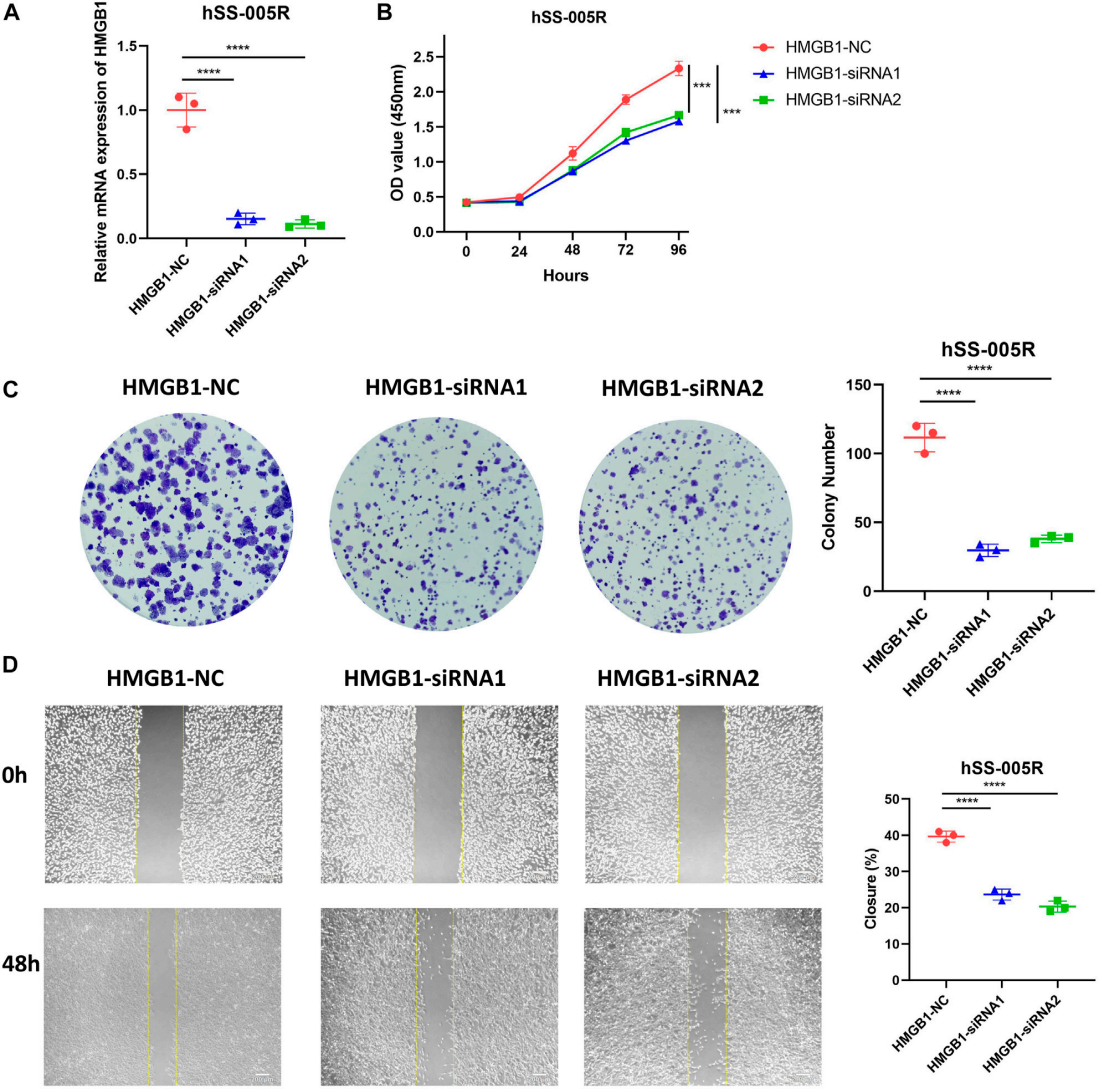
Validation of HMGB1 for promoting malignant biological behaviors of sarcoma cells. **(A)** Assessment of HMGB1 knockdown efficiency in hSS-005R cell lines. Comparison of CCK8 assay **(B)**, clone formation **(C)**, scratch assay **(D)** in hSS-005R cell lines with HMGB1 siRNA and Control siRNA.

In light of the fact that HMGB1 was abnormally upregulated in STS, we proceeded to investigate its roles in STS cell line. In hSS-005R cells transfected with siRNA, the expression of HMGB1 was significantly reduced (Figure 8A). By using the CCK8 assay, Figure 8B showed that inhibition of HMGB1 resulted in a decrease in proliferation rate of hSS-005R. Besides, the ability of hSS-005R cells to form colonies was reduced upon the inhibition of HMGB1 (Figure 8C). In comparison to the control group, siRNA- HMGB1 significantly decreased the migration distance of hSS-005R cells in the scratch test (Figure 8D). The above results suggested that the upregulation of HMGB1 may contribute to the malignant behavior of STS cells, which supports the findings obtained from the bioinformatic analysis.

## Discussion

There are currently limited therapeutic interventions available for STS (Gamboa et al., 2020). In order to be able to identify potential therapeutic targets, many studies analyzed the genomic and transcriptomic features of sarcoma. The processes of NETosis leads to the formation of the NET, which are comprised of modified chromatin and bactericidal proteins derived from the granules and the cytoplasm of neutrophils. There is evidence that the NETs contribute to aggressive mesenchymal phenotypes in gastric cancer (Zhu et al., 2021). The presence of NETosis has been shown to exacerbate cancer by promoting cancer-associated thrombosis and venous thromboembolism (Demers et al., 2012). While many studies have examined the role of NETosis in specific cancer types, there is little research on this topic in STS. Therefore, the current study involved a thorough analysis of NRGs in patients with STS.

According to expression data of 69 NRGs within the TCGA-SARC cohort, this study developed a predictive model for NRGs. By applying Lasso regression analysis and SVM-RFE, 17 genes were further identified as potential predictors. Even though all cancers differ at the molecular level, there are still common mutations or transcriptional regulation features that they share. Our first step was to analyze the molecular characteristics of NRGs at the pan-cancer level. It has been observed that a number of NRGs, including DNASE1, HMGB1 and PIK3CA, undergo gains in SCNV across a variety of cancer types, which was consistent with previous studies (Lin et al., 2011; Ueki et al., 2019; Martell et al., 2020). The PIK3CA has the highest mutation frequency within the TCGA-SARC cohort, suggesting that PIK3CA plays a significant role in STS biology. In several specific cancer types, expression levels of NRGs are associated with prognostic risk, suggesting that their expression can provide insight into prognosis.

The 17 NRGs identified by machine learning methods in STS were further analyzed for their genomic and transcriptomic characteristics. As a result of differential expression, most NRGs have distinct expression patterns that enable differentiation between STS and normal tissues. In our own laboratory, the qPCR and our own sequencing data were used to confirm differential expression in certain NRGs within certain STS cell lines and patient tumor samples. With the advent of scRNA-seq, gene expression patterns within individual cell types can be identified with high resolution and specificity (González-Silva et al., 2020). It is worth highlighting that HMGB1, AKT1 and MTOR exhibited expression within the multiple cell clusters. It has been reported that the mTOR pathway coordinates signaling events within neutrophils after activation, ultimately leading to NETosis (Itakura and McCarty, 2013). Due to the paucity of researches investigating the interaction between NRGs in STS, it is imperative that additional investigations incorporate biological mechanism research would be conducted in the future.

In order to explore the unique features of selected NRGs, we utilized the unsupervised consensus clustering method, which led to the identification of three distinct clusters. By using this approach, it may be possible to reveal conformational nuances that population averages may obscure, thus revealing previously unknown and potentially meaningful patterns (Necchi et al., 2021; Thongprayoon et al., 2022). A comparison of NETosis-related C1 with the other two clusters showed significantly better prognoses. The expression of genes in C1 cluster was markedly increased, which were associated with lysosome function, chemokine signaling pathways, and B cell receptor signaling pathways. Furthermore, the results of ssGSEA analysis indicated that C1 exhibited significantly elevated infiltration of both innate and adaptive immune cells. The majority of cancers have developed mechanisms to evade immune control or immunosurveillance, and infiltration of immune cells and tumor prognosis have been shown to be positively correlated in numerous studies (Fridman et al., 2017; Jiang et al., 2022; Jiang et al., 2023). On the basis of specific NETosis-related clusters, the DEGs were predominantly related to lymphocyte mediated immunity, immune effector regulation, and biotic response regulation. Therefore, NETosis plays a crucial role in regulating immune-related processes (Cedervall and Olsson, 2015). Based on the identification of NETosis-related clusters, this study further identified three NETosis-related subtypes that exhibit distinct prognoses and TME characteristics.

Despite the identification of specific NETosis-related clusters and subtypes based on the TCGA-SARC cohorts, by utilizing systematic approaches, the individual NETosis-related risk needs to be quantified using an accurate method. Therefore, a DEG-based scoring system was further developed. Besides, this study emphasizes the significance of NETosis scoring system in relation to cancer-immunity cycles. Moreover, the findings imply that NETosis may hold regulatory potential over immunotherapy (Wang et al., 2020). With the aim of addressing the lack of specific STS groups treated with immunotherapy, we introduced an independent melanoma cohort receiving a combination therapy of PD-1 and CTLA-4 blockade. Our results have confirmed the strength of the NETosis score, despite the need for further prospective studies of STS with immunotherapy. In studies of NETosis-related genes in head and neck squamous cell carcinoma and bladder cancer, NETosis has been found to play a key role in the progression of these cancers and can be used for prognostic prediction. Besides, the NETosis scoring system could serve as an efficient tool for predicting the prognosis and response to immunotherapy in individuals with STS.

To summarize, the current study provides the first comprehensive analysis of NETosis-related gene patterns in STS. As a result of our study, we have shed light on the intricate profiling and cross-talk of NRGs in pan-cancer level well as STS cell lines, elucidating its key role in tumor biology. With the model of NETosis score, personalized therapeutic approaches can be enhanced and optimized in patients with STS. In a broader sense, this research highlights the important interplay between NRGs, which provides new insights into treatment strategies for STS patients.

## Data Availability

Publicly available datasets were analyzed in this study. This data can be found here: UCSC Xena (https://xena.ucsc.edu/) and GEO database (https://www.ncbi.nlm.nih.gov/geo/) with accession Nos GSE17118, GSE30929, GSE131309, and GSE198568.

## Supplementary material

SUPPLEMENTARY FIGURE S1 Analysis of single-cell expression patterns of NRGs across different cell types in soft-tissue sarcoma.

SUPPLEMENTARY FIGURE S2 Identification of NETosis-related clusters. Consensus clustering based on NRGs (K=2-6). **(F)** Consensus cumulative distribution function (CDF) plot based on SRGs. **(G)** GSVA revealing distinct enriched pathways among NETosis-related clusters.

SUPPLEMENTARY FIGURE S3 Identification of NETosis-related subtypes. Consensus clustering based on DEGs associated with NETosis-related clusters (*K* = 2–6). (F) The CDF plot based on DEGs associated with NETosis-related clusters. **(G)** GSVA revealing distinct enriched pathways among NETosis-related subtypes.

SUPPLEMENTARY FIGURE S4 Prognostic analysis of the TCGA-SARC cohort by subgroups based on NETosis score. The Kaplan-Meier analysis of low and high NETosis score groups with different ages , gender **(C, D)**, sites **(E, F)** and histology **(G–K)**.
