# Supplementary figures and images for: Deciphering the role of NETosis-related signatures in the prognosis and immunotherapy of soft-tissue sarcoma using machine learning
Source: Front Pharmacol. 2023 Jun 20;14:1217488. doi: 10.3389/fphar.2023.1217488 (PMC10318157; doi:10.3389/fphar.2023.1217488)

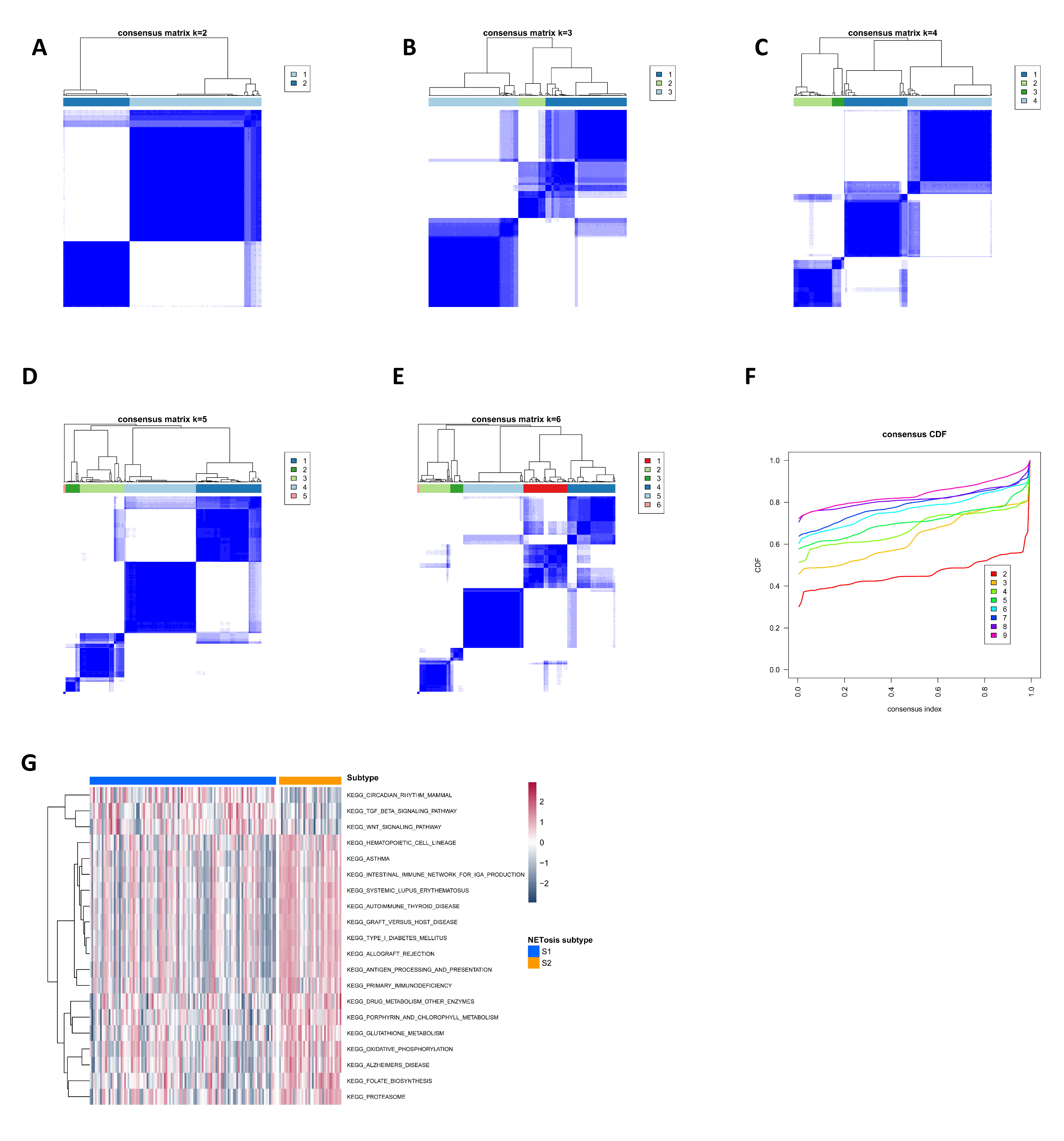

Supplement: Supplementary file 1 [file Image3.JPEG]

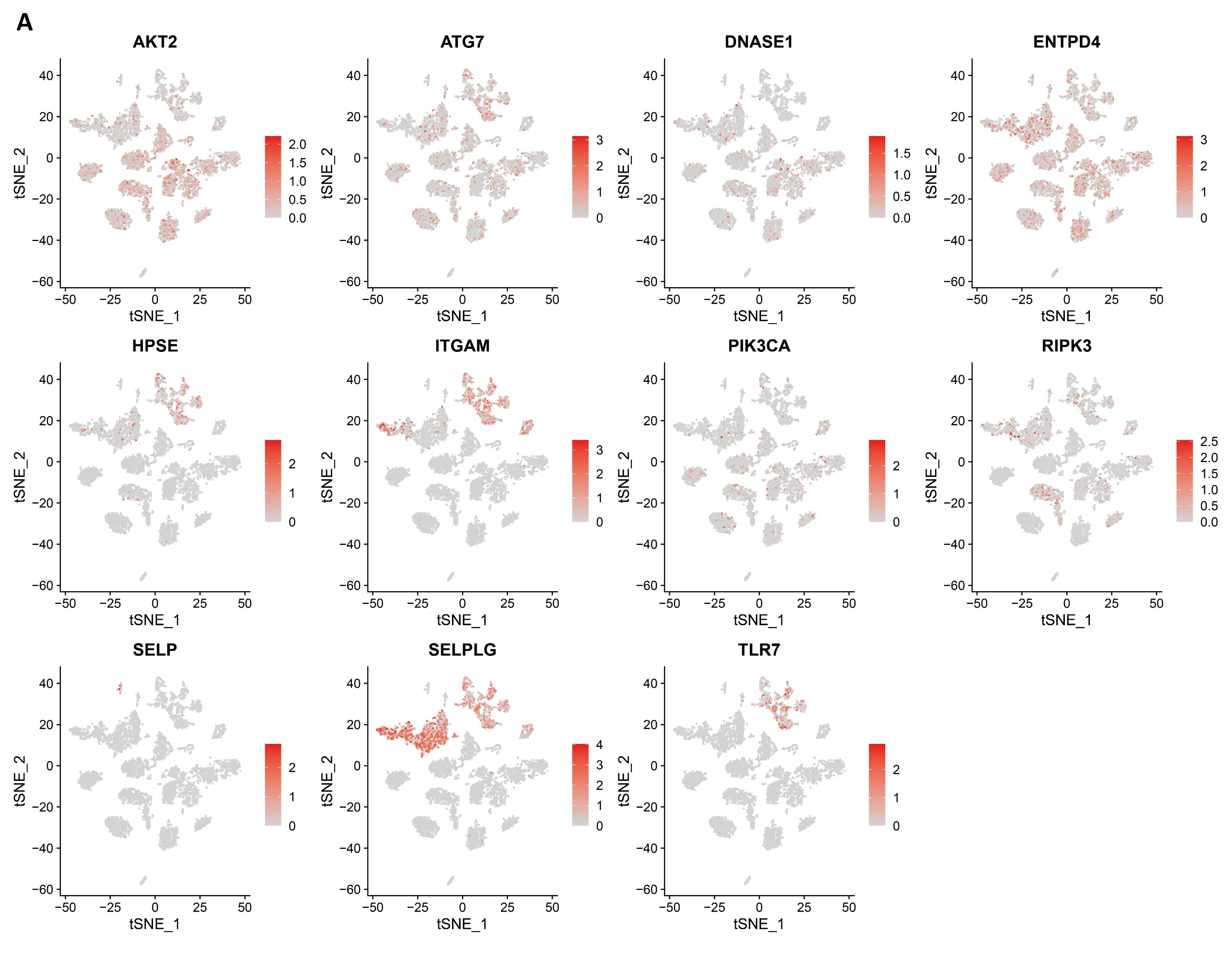

Supplement: Supplementary file 2 [file Image1.JPEG]

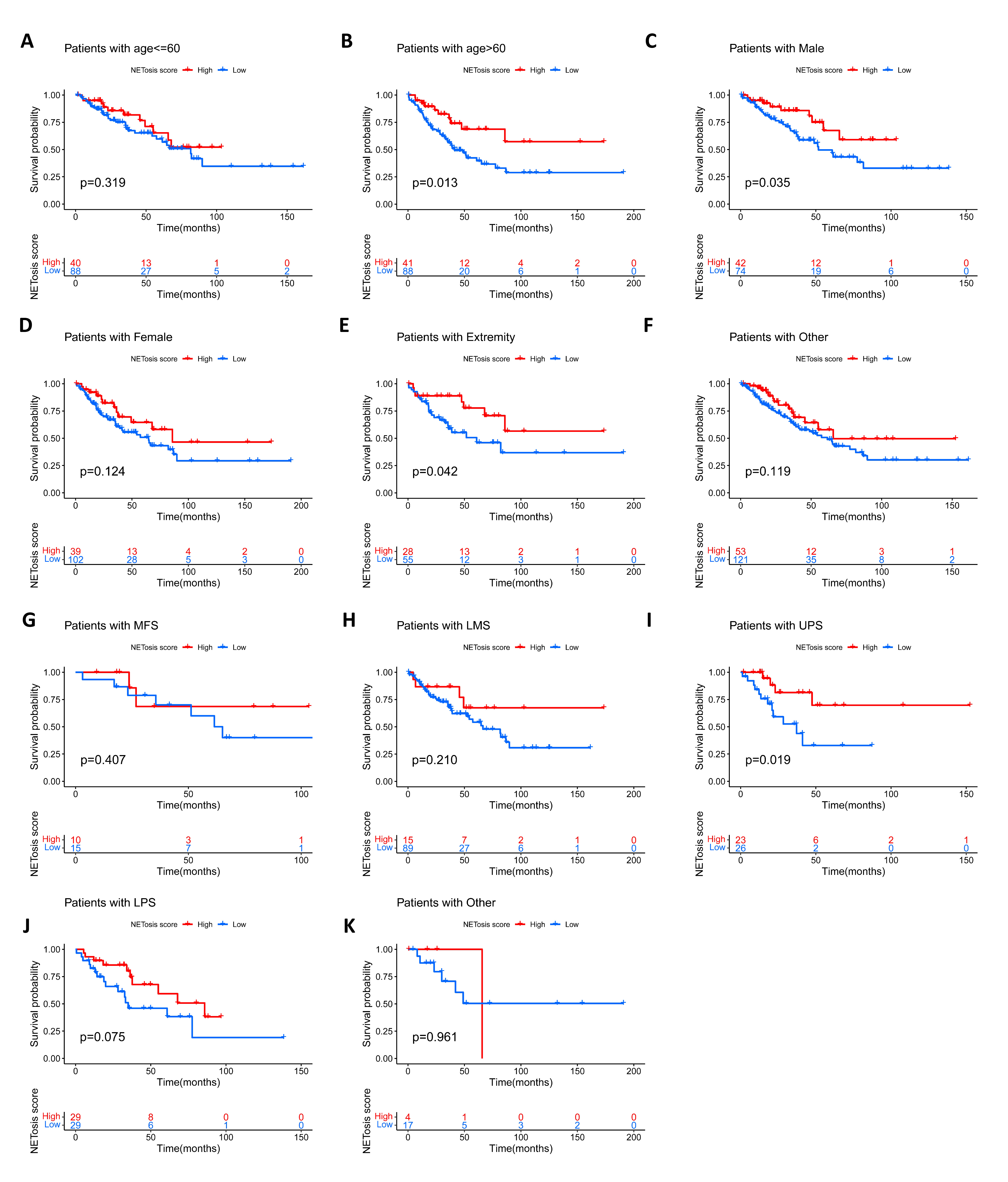

Supplement: Supplementary file 3 [file Image4.JPEG]

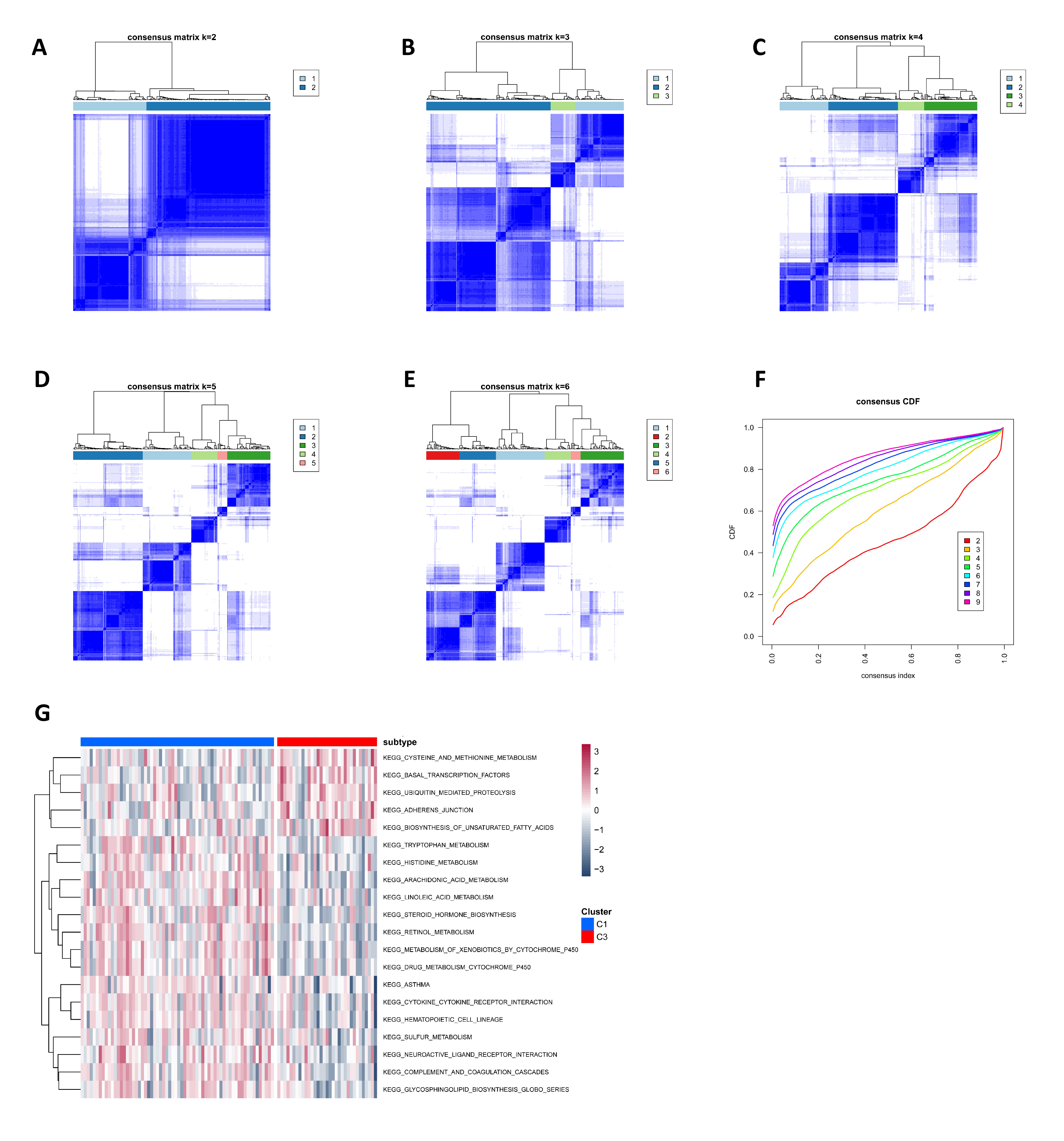

Supplement: Supplementary file 4 [file Image2.JPEG]
